# Triple NF-kB binding sites and LTR sequence similarities in HIV-1C isolates irrespective of helminth co-infection

**DOI:** 10.1186/1756-3305-7-204

**Published:** 2014-04-29

**Authors:** Andargachew Mulu, Melanie Maier, Uwe Gerd Liebert

**Affiliations:** 1 Institute of Virology, Leipzig University, Johannisallee 30, Leipzig 04103, Germany; 2 Department of Microbiology, College of Medicine and Health Sciences, University of Gondar, Gondar, Ethiopia

**Keywords:** NF-κB, LTR sequences, HIV-1 C/C’ subclusters, Helminths, HIV viral load

## Abstract

**Background:**

Helminth infections as well as structural alternations in the long-terminal repeat (LTR) regions of HIV-1 are known to contribute to elevated HIV RNA level and enhance HIV-1 diseases progression. However, the impact of helminths infections on the occurrences of triple NF-κB and genetic variability in LTR region of HIV-1C isolates is not known. We aimed to examine the presence of genetic variability in the LTR region of HIV-1C isolates during chronic HIV-helminth co-infection.

**Methods:**

HIV-1C infected Ethiopians with (n = 22) and without (n = 20) helminth infection were included. The LTR region of HIV was amplified and sequenced. Sequences were aligned with reference set from the Los Alamos HIV database. Phylogenetic analysis and frequency of polymorphic changes was performed by the neighbour-joining method using Geneious Basic software.

**Results:**

All LTR sequences from patients with or without of helminth co-infection clustered with HIV-1 subtype C with two distinct subclusters (C and C’). The enhancer element was found to have three copies of 10-base pair binding sites for NF-κBs which is an evidence for predominance of triple NF-κB sites (94%) in HIV-1C isolates irrespective of helminths co-infection and subclusters. Moreover, irrespective of helminth co-infection and C/C’ subclusters high sequences similarity in LTR was observed. There was no significant difference in plasma HIV RNA level between HIV-1 C and C’ subclusters.

**Conclusions:**

Despite the small sample size, the predominance of triple NF-κB binding sites and high sequence similarities in LTR region irrespective of helminths infection suggest the natural occurrence of the three NF-κB binding sites in HIV-1C isolates without the influence of secondary infection. Thus, the higher HIV-1C viraemia in helminth co-infected individuals is more likely a result of immune activation rather than LTR sequence variation. Moreover, the lack of significant difference in plasma HIV RNA level between HIV-1 C and C’ subcluster may show the lack of functional differences among the two groups.

## Background

According to UNAIDS report, half of the people infected with HIV-1 in sub Saharan Africa are estimated to be co-infected with helminths [1] which may accelerate the course of HIV by augmenting viral replication [2,3]. Immunologic evidence on the mechanisms by which chronic helminth infection may facilitate HIV-1 replication in co-infected individuals is well documented [4]. It is suggested that HIV/helminths co-infected patients experience a shift from a Th1 to a predominantly Th2 response [4-6]. Consecutively, it has been speculated that chronic immune activation and the presence of a dominant Th2 cytokine environment may increase the risk of acquiring HIV infection [6,7]. The chronic immune activation has been proposed as the main cause for elevated plasma HIV RNA level reported among sub Saharan Africa patients [8]. The role of intestinal helminth co-infection and deworming on HIV viral load among acute sero-converters is controversial [9-12]. During chronic HIV-1 infection there is a sustained and dramatic immune activation characterized by elevated plasma levels of TNF-alpha, IL-1, and IL-6, which is directly correlated with plasma HIV RNA levels [13]. We have recently observed higher HIV-1C viraemia in chronically helminthic co-infected patients, which was significantly reduced after 12 weeks of deworming [14].

Indeed, viral factors and their interaction with host factors also indicate the risk of HIV disease progression, elevated plasma HIV RNA level, and higher HIV transmission rate reported among sub Saharan African patients [13,15]. For example, HIV-1 long-terminal repeat (LTR) is known to regulate viral gene expression by interacting with multiple viral and host factors. Reports on the contribution of structural alterations in the LTR region to disease progression and higher HIV transmission rate are controversial. Intracellular parasitic infection like *Leishmania* was found to be a potential activator for HIV replication and potent inducer of LTR transcription and viral replication *in vitro*. Moreover, the transcription factor NF-κB in the enhancer region of the LTR is activated through infection with viral and other pathogens independently [16]. Activation of HIV-1 LTR transcription in host cells through complex biochemical pathways involving the participation of transcriptional factor NF-κB has been observed during leishmania parasite co-infection *in vitro*[16]. The sequence of the enhancer element varies among HIV-1 subtypes [17]. Triple NF-κB sequences have been described in most HIV-1C isolates [18-20] and believed to influence the viral fitness and higher replication and transmission. However, the relationship between the occurrences of triple NF-κB in most HIV-1C isolates and helminths co-infection is not known. During chronic HIV-1 infection with helminth co-infection, the ensuing immunological imbalance could conceivably lead to increased expression of co-receptor and transcriptional factors resulting in higher HIV RNA level and faster disease progression which may in turn result in active up-regulation of HIV LTR. Such functional shifts could be a result of structural alteration on the transcriptional factor NF-κB. We hypothesise that the higher HIV RNA level in helminths co-infected patients may also be associated with sequence alterations in LTR region [14]. Thus, the aim of the current study was to examine the impact of helminths on genetic variability on LTR region of HIV-1 during chronic HIV-helminth co-infection.

## Methods

This study was aimed to investigate the effect of helminths infection on the presence of alterations in regions and motifs of LTR during chronic HIV-helminths infection. The details of patient inclusion were described previously [14]. Briefly, symptomatic HIV-infected persons above 18 years of age, seeking treatment and willing to participate were evaluated with a standardized form at enrolment. Patients were excluded for the following reasons or conditions: pregnancy, treatment with single dose nevirapine for prevention of mother-to-child transmission of HIV or any other antiretroviral therapy (ART), known diabetes, hypertension, epilepsy, liver, cardiac and renal diseases, genital ulcer or active tuberculosis at enrolment. T-cell count was made using flow cytometer (FACSCount, Becton Dickinson, San Jose, California, USA) following the manufacturer’s protocol. Plasma HIV-1 RNA level was determined by quantitative real time PCR (Abbott m2000rt instrument, Abbott Molecular, Des Plaines, IL, USA). The lower detection limit of the assay was 40 copies/ml (1.6 log_10_ RNA copies/ml).

The LTR region was amplified by nested PCR using a published protocol [18]. Nucleic acid sequencing was performed using the inner primers and the ABI prism 310 Dye Terminator Cycle Sequencing Kit according to the manufacturing instruction (Applied Biosystems, Foster City, CA, USA). Sequences were aligned with reference set from the Los Alamos HIV database (http://www.hiv.lanl.gov). Phylogenetic analysis was performed by the neighbour-joining method with 1,000 bootstrap replicates under Kimura’s two-parameter correction using Geneious Basic software (http://www.geneious.com). The frequency of polymorphic changes (number of nucleotide changes found in each consensus sequence with respect to ETH2220 reference sequence divided by the total number of nucleotides sequenced) was calculated for enhancer, promoter regions and TRA elements of the LTR regions relevant for transcription.

## Results

**Figure 1:**
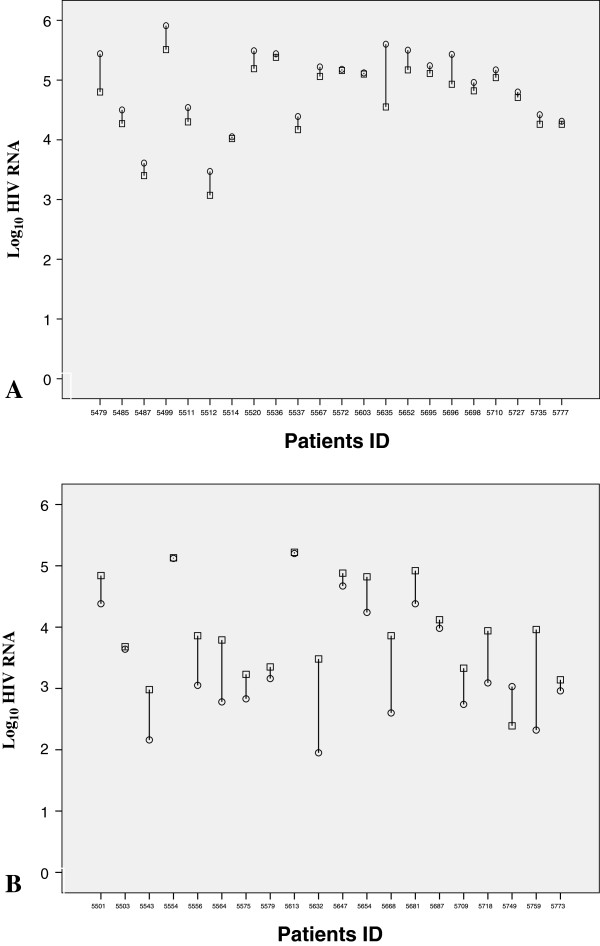
Dynamics of plasma HIV RNA level of each patient with (A) and without (B) helminths infection at baseline (circles) and after 12 weeks of administration of antihelminthic drugs (squares).

The baseline demographic data (Table 1), baseline differences in plasma HIV RNA level and T cell counts and the impact of helminth co-infection on HIV-1 viraemia and its dynamics before and 12 weeks after deworming (Figure 1) were described previously [14]. The mean plasma HIV RNA level at baseline was 4.30 ± 1.09 log_10_ RNA copies/ml. There were no difference in plasma HIV RNA level (P = 0.7) among females and males. However, plasma HIV RNA level was significantly higher (4.83 ± 0.9 versus 3.95 ± 1.0 log_10_ HIV RNA copies/ml) in individuals co-infected with intestinal parasites than in those without (Table 1). There was a mean decrease in plasma HIV level (−0.3 log_10_ ± 0.83) after successful treatment of helminths, but an increase in HIV load in patients that had no helminth coinfection (Figure 1) in parallel with significant reduction in CD4^+^ T cells.

**Table 1:** Characteristics of the study participants at enrolment

**Variables**	**With helminths (n = 87)**	**Without helminths (n = 133)**	***P value***
Sex			
Male	42	50	n.a.
Female	45	83	n.a.
Age (Mean + SD)	31.4 ± 9^a^	33.7 ± 8	n.a.
log_10_ HIV RNA (copies/ml)	4.83 ± 0.85	3.95 ± 0.96	<0.001
CD4^+^ T cell/mm^3^	214 ± 142	212 ± 160	0.944
CD8^+^ T cell/mm^3^	970 ± 566	893 ± 427	0.035

n.a: Not applicable; ^a^Mean ± SD.

**Figure 2:**
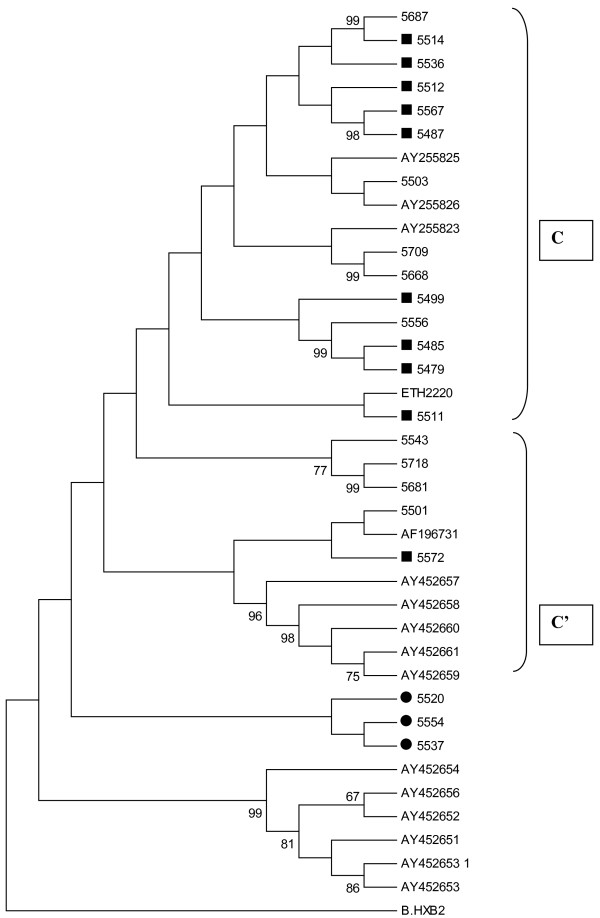
**Phylogenetic tree of LTR sequences from chronically infected patients with and without helminths coinfection (Current isolates are designated by 4 digit numbers and the rest are reference sequences).** The two genotypes cocirculating are indicated as C and C’. **Keys**: Sequences with Black Square (■) are from patients with chronic helminths infection and with Black circles (●) are isolates which could not be classified in either one of the two subclusters.

Phylogenetic analysis revealed that all LTR sequences from patients with and without helminths co-infection were clustered within HIV-1 subtype C (Figure 2) concordant with the respective protease and reverse transcriptase sequences of HIV-1 genome [21]. However, two distinct sub-clusters (C/C’) with bootstrap values of less than 65% were observed (Figure 2). The C subclusters which is related to the Ethiopian reference strain (ETH2220) were higher than the C’ subclusters which possesses phylogenic links to the strains circulating in southern Africa (63.6 versus 22.7%). Three of the 22 (13.6%) isolates could not be classified in either one of the two subclusters. Irrespective of helminths infection and CD4^+^ T cell count, there was no significant difference in plasma HIV RNA level at baseline and 12 weeks after deworming (4.42 ± 0.6 versus 4.33 ± 0.7 log_10_ HIV RNA copies/ml; 4.12 ± 0.8 versus 4.22 ± 1.0 log_10_ HIV RNA copies/ml) between in HIV-1 C and C’ subclusters, respectively. This observation remained the same after CD4^+^ T cell counts was stratified as low (<200 cells/mm^3^), medium 201–350 cells/mm^3^) and high (>350 cell/mm^3^).

**Figure 3:**
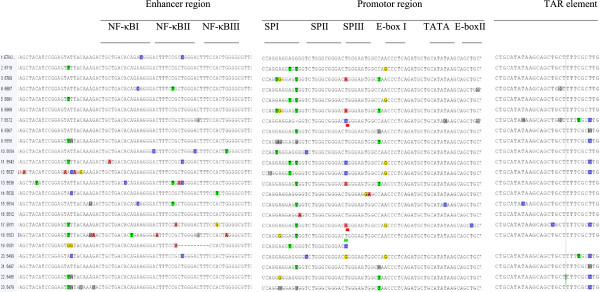
**Sequences of most enhancer, promoter region and LTR element of isolates with and without helminths coinfection with respect to ETH2220 consensus reference.** Sequences of 5572, 5567, 5537, 5536, 5520, 5514, 5512, 5511, 5499, 5487, 5485 and 5479, are patients with chronic helminths infection. Sequences of 5543, 5718, 5681, 5501 and 5572 are HIV-1C’ subclusters. Keys: NF-KB-Nuclear factor Kappa B; TAR element-Transcription trans-activator.

The enhancer element was found to have three copies of 10-base pair binding sites for NF-κB which is an evidence for predominance of triple NF-κB sites (94%) of the isolates with only one isolate having two NF-κB sites (with deletion of 11 nucleotides). No significant differences were observed in the mutation/polymorphic frequency in most LTR regulatory sites among patients with and without helminths co-infection (Table 2, Figure 3) and among C and C’ subclusters. Briefly, the core region of the LTR which is composed of the TATAA box (29–24 nucleotides) upstream of the transcriptional start site, and specificity protein (Sp) binding sites which are three tandem GC-rich binding sites interacting with transcription factors SpI to SpIII were the most conserved regions. Moreover, SpII site of the promoter region was identical in both groups of patients. The TATAA box, which binds TATAA-binding protein in association with a number of other proteins was the second most identical region. Six closely related monophyletic groups with bootstrap value of 99% (Figure 2) were also observed which is concordant with their respective pol genome [21].

**Table 2:** LTR genetic variability on transcriptional and regulatory sites in HIV-1 subtype C chronically infected individuals with and without helminths coinfections

**Variables**	**Mutation frequency (x10**^**−2**^**)**^**a**^									
	**Enhancer region**			**Promotor region**					**TAR element**	
	**NF-κBI**	**NF-κBII**	**NF-κBIII**	**SPI**	**SP II**	**SP III**	**E-box I**	**TATA box**	**E-box II**	**TAR**
With helminths infection	8.5	12.4	2.6	12.4	0	8.8	2.5	3.1	1.7	7.1
Without helminths infection	9.3	11.3	1.6	14.1	0	5.3	5.0	2.7	1.7	7.7

^a^Polymorphic changes are calculated by dividing the number of changes with respect to ETH2220 sequence by total number of nucleotides sequenced in each region; NF-κB-Nuclear factor Kappa B; TAR element-Transcription trans-activator.

## Discussion

This is the first study, which examines the genetic variability of HIV-1C LTR region among chronically infected patients with and without helminths co-infection. The findings of LTR high genetic similarity in most of the regulatory sites irrespective of helminths co-infections may suggest the compensation of immunological imbalance [4,13,16,18] which could be induced by both chronic HIV and helminths infections. Sequence inconsistency in the LTR binding sites (the promoter, enhancer, modulatory and TAR regions) have been observed in other subtypes. Nevertheless, it remains unclear for the majority of the clades that whether the observed sequence differences could influence the replicative capacity of the subtypes [16,17]. The predominance of triple NF-κB sites in the current Ethiopian isolates is similar with previous studies from Africa and the rest of the world where subtype C is predominately circulating [17-20]. The occurrence of triple NF-κB sites with and without helminths infection shows the lack of association between helminths infection and presence of the third NF-κB sites; and is an additional evidence for the natural occurrence of triple NF-κB sites in HIV-1C isolates [17,19], irrespective of co-infection. Thus, the biological influence of helminths could not directly determine genetic variability or similarity, suggesting that the observed high level of HIV-1C viraemia during chronic HIV-helminth co-infection [4,6,9-12,14] is due to the immune activation [4,8]. Although deletion of one or two NF-κB sites leads to substantially decreased replication levels [20], the subject 5627 (Figure 3) in the current study with deletion of the second NF-κB sites (11 nucleotides) had comparable HIV RNA level with the rest of the subject. Indeed, the function of this additional site may be altered during co-infection because of the immune dysregulation or activation.

Previous studies in Ethiopia showed the presence of two distinct subtypes C subclusters (C and C’) based on phylogenetic analysis of the *env* region [22-27] and the increasing of subcluster C’ overtime [23] although the epidemic of both strains began around the same time [25]. The separate cluster of the LTR sequences from these chronically infected patients in the current study compared with Ethiopian reference isolates (ETH2220) strengthen previous studies that demonstrated that the Ethiopian subtype C had a genetic subcluster designated C’ in the *LTR* regions [22-27]. The genetic subclusters may indicate a different introduction of HIV in to Ethiopia [23] and diversity of the circulating HIV-1 C subtypes in the country. Although, the prevalence of C’ was increasing from 48% in 1988 to 70% in 1997 based on *env* gene [23,26], indicating that C’ viruses may be spreading faster in Ethiopia, we found a higher C subcluster compared with C’ similar with previous report from central Ethiopia [27] indicating that when considering the LTR sequence the one from the C genotype may be advantageous in northwest Ethiopia. Unlike previous report [26], in the current study lack of significant difference in plasma HIV RNA level between HIV-1 C and C’ subcluster was observed and may show the lack of functional differences among the two groups. The 63.6% HIV-1 C subcluster in northwest Ethiopia is additional epidemiological evidence which demonstrates that subcluster C has deep rooted and is the predominant genotype currently circulating in northwest Ethiopia.

## Conclusions

Despite the small sample size, the predominance of triple NF-κB sites and high sequence similarities in most LTR regions irrespective of helminths co-infection suggest the natural occurrence of additional NF-κB sites in HIV-1C isolates without the influence of helminths infection. Thus, the higher HIV-1C viraemia in helminth co-infected individuals is more likely a result of immune activation rather than LTR sequence variation. The lack of significant difference in plasma HIV RNA level between HIV-1 C and C’ subcluster may show the lack of functional differences among the two groups. However, it needs further studies to describe the role of HIV-1 C/C’ genetic variants in HIV-1 immunopathogenesis.

## Competing interests

The authors declare that they have no competing interest.

## Authors’ contributions

All authors participated in the design, analysis, and interpretation of the study and writing the manuscript. All authors read and approved the final manuscript.

## References

[B1] UNAIDS Epidemic Update 2010 http://www.unaids.org/globalreport/

[B2] Bentwich Z Weisman Z Grossman Z Pathogenesis of AIDS in Africa-lessons from the Ethiopian immigrants in Israel Immunologist 1997 5 211 216

[B3] Lawn SD Butera ST Folks TM Contribution of immune activation to the pathogenesis and transmission of human immunodeficiency virus type 1 infection Clin Microbiol Rev 2011 14 753 757 11585784 10.1128/CMR.14.4.753-777.2001 PMC89002

[B4] Bentwich Z Maartens G Torten D Bar-Yehuda Kalikovich A Concurrent infections and HIV pathogenesis AIDS 2071–2081 12000 4 10.1097/00002030-200009290-00002 11061647

[B5] Kassu A Tsegaye A Wolday D Petros B Aklilu M Sanders EJ Fontanet AL Van Baarle D Hamann D De Wit TF Role of incidental and/or cured intestinal parasitic infections on profile of CD4+ and CD8^+^ T cell subsets and activation status in HIV-1 infected and uninfected adult Ethiopians Clin Exp Immunol 2003 132 113 119 10.1046/j.1365-2249.2003.02106.x 12653845 PMC1808681

[B6] Borkow G Bentwich Z HIV and helminth co-infection: is deworming necessary? Parasite Immunol 2000 28 605 612 17042932 10.1111/j.1365-3024.2006.00918.x

[B7] Morgan D Whitworth J The natural history of HIV-1 infection in Africa Nat Med 2001 7 143 145 10.1038/84564 11175832

[B8] Bentwich Z Kalinkovich A Weisman Z Borkow G Beyers N Beyers A Can eradication of helminthic infections change the face of AIDS and tuberculosis? Immunol Today 1999 20 485 487 10.1016/S0167-5699(99)01499-1 10529774

[B9] Wolday D Mayaan S Mariam ZG Berhe N Seboxa T Britton S Galai N Landay A Bentwich Z Treatment of intestinal worms is associated with decreased HIV plasma viral load J Acquir Immune Defic Syndr 2002 31 56 62 10.1097/00126334-200209010-00008 12352151

[B10] Modjarrad K Zulu I Redden DT Njobvu L Lane HC Bentwich Z Vermund SH Treatment of intestinal helminths does not reduce plasma concentrations of HIV-1 RNA in coinfected Zambian adults J Infect Dis 2005 192 1277 1283 10.1086/444543 16136473 PMC2730764

[B11] Hosseinipour MC Napravnik S Joaki G Gama S Mbeye N Banda B Martinson F Hoffman I Cohen MS HIV and parasitic infection and the effect of treatment among adult outpatients in Malawi J Infect Dis 2007 195 1278 1282 10.1086/513274 17396996

[B12] Walson JL Herrin BR John-Stewart G Deworming helminth co-infected individuals for delaying HIV disease progression Cochrane Database Syst Rev 2009 3 CD006419 doi:10.1002/14651858.CD006419.pub3 19588389 10.1002/14651858.CD006419.pub3 PMC2871762

[B13] Graziosi C Soudeyns H Rizzardi PG Immunopathogenesis of HIV infection AIDS Res Hum Retroviruses 1998 14 S135 S142 9672230

[B14] Mulu A Maier M Liebert UG Deworming of intestinal helminths reduces HIV-1 subtype C viremia in chronically co-infected individuals Int J Infect Dis 2013 17 e897 e901 10.1016/j.ijid.2013.03.022 23688549

[B15] Alemu A Shiferaw Y Addis Z Mathewos B Birhan W Effect of malaria on HIV/AIDS transmission and progression Parasites & Vectors 2013 6 18 doi:10.1186/1756-3305-6-18 10.1186/1756-3305-6-18 23327493 PMC3564906

[B16] Olivier M Badaró R Medrano FJ Moreno J The pathogenesis of Leishmania/HIV co-infection: cellular and immunological mechanisms Ann Trop Med Parasitol 2000 97 S79 S98 10.1179/000349803225002561 14678636

[B17] Arellano Eva R Soriano V Alcami J Holguin A A new finding on the transcription regulation across different subtypes AIDS Rev 2006 8 9 16 16736947

[B18] De Baar MP De Ronde A Berkhout B Cornelissen M Van Der Horn KH Van Der Schoot AM De Wolf F Lukashov VV Goudsmit J Subtype-Specific Sequence Variation of the HIV Type 1 Long Terminal Repeat and Primer-Binding Site AIDS Res Hum Retroviruses 2000 16 499 504 10.1089/088922200309160 10772536

[B19] Jeeninga RE Hoogenkamp M Armand-Ugon M De Baar M Verhoef K Berkhout B Functional Differences between the Long Terminal Repeat Transcriptional Promoters of Human Immunodeficiency Virus Type 1 Subtypes A through G J Virol 2000 74 3740 3751 10.1128/JVI.74.8.3740-3751.2000 10729149 PMC111883

[B20] Bachu M Yalla S Asokan M Verma A Neogi U Sharma S Murali RV Mukthey AB Bhatt R Chatterjee S Rajan RE Cheedarla N Yadavalli VS Mahadevan A Shankar SK Rajagopalan N Shet A Saravanan S Balakrishnan P Solomon S Vajpayee M Satish KS Kundu TK Jeang KT Ranga U Multiple NF-κB sites in HIV-1 subtype C LTR confer superior magnitude of transcription and thereby the enhanced viral predominance J Biochem 2012 http://www.jbc.org/cgi/doi/10.1074/jbc.M112.397158 10.1074/jbc.M112.397158 PMC3531786 23132857

[B21] Mulu A Lange T Liebert UG Maier M Clade homogeneity and Pol gene polymorphisms in chronically HIV-1 infected antiretroviral treatment naive patients after the roll out of ART in Ethiopia BMC Infect Dis 2014 14 158 Doi: 10.1186/1471-2334-14-158 10.1186/1471-2334-14-158 24655349 PMC3976149

[B22] Kassu A Fujino M Matsuda M Nishizawa M Ota F Sugiura W Molecular epidemiology of HIV-1 in treatment naive patients in North Ethiopia AIDS Res Hum Retroviruses 2007 23 564 568 10.1089/aid.2006.0270 17451346

[B23] Pollakis G Abebe A Kliphuis A De Wit TF Fisseha B Tegbaru B Tesfaye G Negassa H Mengistu Y Fontanet AL Cornelissen M Goudsmit J Recombination of HIV type 1C (C’/C") in Ethiopia: Possible link of EthHIV-1C’ to subtype C sequences from the high-prevalence epidemics in India and Southern Africa AIDS Res Hum Retroviruses 2003 19 999 1008 10.1089/088922203322588350 14678607

[B24] Abebe A Pollakis G Fontanet AL Fisseha B Tegbaru B Kliphuis A Tesfaye G Negassa H Cornelissen M Goudsmit J Rinke De Wit TF Identification of a genetic subcluster of HIV type 1 subtype C (C’) widespread in Ethiopia AIDS Res Hum Retroviruses 2000 16 1909 1914 10.1089/08892220050195865 11118076

[B25] Abebe A Lukashov VV Rinke De Wit TF Fisseha B Tegbaru B Kliphuis A Tesfaye G Negassa H Fontanet AL Goudsmit J Pollakis G Timing of the introduction into Ethiopia of subcluster C’ of HIV type 1 subtype C AIDS Res Hum Retroviruses 2001 17 657 661 10.1089/088922201300119770 11375063

[B26] Ayele W Mekonnen Y Messele T Mengistu Y Tsegaye A Bakker M Berkhout B Dorigo-Zetsma W Wolday D Goudsmit J Coutinho R De Baar M Paxton W Pollakis G Differences in HIV Type 1 RNA plasma load profile of closely related cocirculating Ethiopian subtype C strains: C and C’ AIDS Res Hum Retroviruses 2010 26 805 813 10.1089/aid.2009.0152 20624072

[B27] De Baar M Abebe A Kliphuis A Tesfaye G Goudsmit J Pollakis G HIV Type 1 C and C’ subclusters based on long terminal repeat sequences in the Ethiopian HIV type 1 subtype C epidemic AIDS Res Hum Retroviruses 2002 19 917 922 14601586 10.1089/088922203322493094

